# Biochemical and Functional Comparisons of *mdx* and *Sgcg* ^−/−^ Muscular Dystrophy Mouse Models

**DOI:** 10.1155/2015/131436

**Published:** 2015-05-03

**Authors:** Nathan W. Roberts, Jenan Holley-Cuthrell, Magdalis Gonzalez-Vega, Aaron J. Mull, Ahlke Heydemann

**Affiliations:** ^1^Department of Physiology and Biophysics, The University of Illinois at Chicago, Chicago, IL 60612, USA; ^2^Center for Cardiovascular Research, The University of Illinois at Chicago, Chicago, IL 60612, USA

## Abstract

Mouse models have provided an essential platform to investigate facets of human diseases, from etiology, diagnosis, and prognosis, to potential treatments. Muscular dystrophy (MD) is the most common human genetic disease occurring in approximately 1 in 2500 births. The *mdx* mouse, which is dystrophin-deficient, has long been used to model this disease. However, this mouse strain displays a rather mild disease course compared to human patients. The *mdx* mice have been bred to additional genetically engineered mice to worsen the disease. Alternatively, other genes which cause human MD have been genetically disrupted in mice. We are now comparing disease progression from one of these alternative gene disruptions, the *γ*-sarcoglycan null mouse *Sgcg* ^−/−^ on the DBA2/J background, to the *mdx* mouse line. This paper aims to assess the time-course severity of the disease in the mouse models and determine which is best for MD research. The *Sgcg* ^−/−^ mice have a more severe phenotype than the *mdx* mice. Muscle function was assessed by plethysmography and echocardiography. Histologically the *Sgcg* ^−/−^ mice displayed increased fibrosis and variable fiber size. By quantitative Evan's blue dye uptake and hydroxyproline content two key disease determinants, membrane permeability and fibrosis respectively, were also proven worse in the *Sgcg* ^−/−^ mice.

## 1. Introduction

A satisfactory mouse model is required to fight the devastating effects of muscular dystrophy (MD). The mouse model must mimic the human disease in etiology, pathology, and potential therapy responses to be optimally useful. In the experiments presented in this paper we directly compared the progressive pathology of two MD mouse models side-by-side to identify the most severe and therefore the most human-similar and useful model. We produced comparative time-courses of disease progression of the* mdx* and *γ*-sarcoglycan null mice (official nomenclature, *Dmd* ^*mdx*^, and* Sgcg* ^−/−^ D2) using quantitative membrane permeability and fibrosis assessments, plethysmography, and echocardiography.

**Figure 1:**
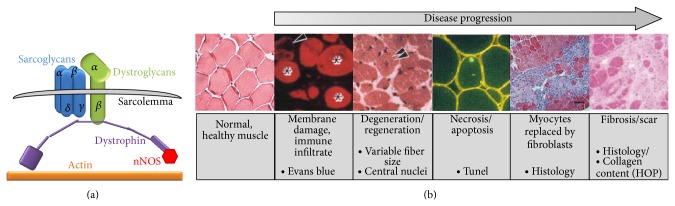
The dystrophin glycoprotein complex and muscular dystrophy pathology in mice. (a) Muscular dystrophy can occur from mutations in the genes that form the dystrophin glycoprotein complex. (b) Representative images demonstrating disease progression of muscular dystrophy in a mouse model.

**Figure 2:**
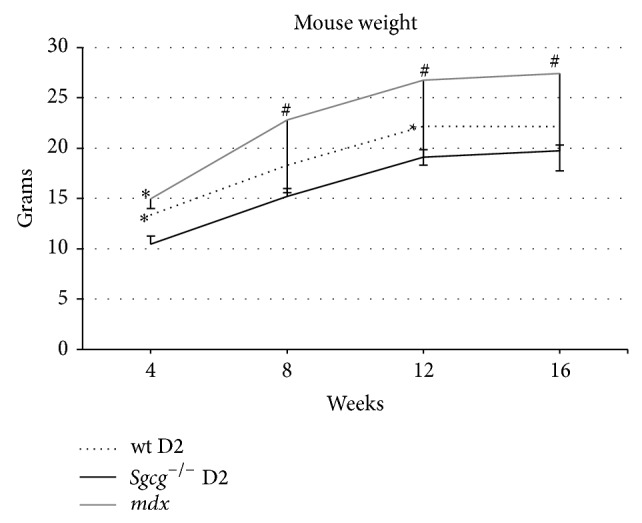
Consistent with severe pathology the* Sgcg* ^−/−^ D2 mice weighed less that their wild type D2 controls throughout the 16 weeks. Wild type D2 mice weighed significantly more than the* Sgcg* ^−/−^ D2 mice at 4 and 12 weeks. ^∗^ *P* < 0.05 significance by Student's* t*-test versus* Sgcg* ^−/−^ D2 animals. *n* = 4–20.

Muscular dystrophy in humans is caused by mutations in a number of genes. The genes can be divided into 4 loosely aligned functional classes (1) dystrophin glycoprotein complex (DGC), which includes dystrophin and the sarcoglycans (Figure 1(a)), (2) extracellular matrix genes, (3) nuclear membrane genes, and (4) signaling molecules. By far the most frequent mutations are found in the DGC component dystrophin, these mutations cause Duchenne (DMD) or Becker muscular dystrophies in humans and* mdx* in mice. Mutations in the *γ*-sarcoglycan gene of the DGC cause limb girdle muscular dystrophy type 2C (LGMD-2C) in humans and are designated* Sgcg* ^−/−^ in mice.

The pathology and treatments of human DMD and LGMD-2C are severe and similar [1]. After the initial disruption of the DGC, disease progression follows a very similar course (Figure 1(b)): (1) membrane permeability followed by mislocalized calcium, nitric oxide, and other signaling moieties, (2) repeated rounds of degeneration and regeneration, (3) inflammation, (4) necrosis, and (5) replacement with myofibroblasts and scar tissue leading to functional decline. These events do not occur in strict sequence and in a single muscle all of these events are occurring at the same time. The* mdx* and* Sgcg* ^−/−^ mice also display the same disease progression, but as we will demonstrate at different severity levels.

As DMD is the most common form of human muscular dystrophy, the dystrophin deficient* mdx* mouse is the most commonly used animal model to represent this disease. The* mdx* mutation was spontaneously identified in a colony of C57BL/10 mice [2]. It was later identified to be in the dystrophin gene [3], which was also identified to be causative for DMD [4]. The originally mutated mouse was identifiable because the dystrophin gene is on the X-chromosome and the original male could be assessed in the hemizygous state.

However, the* mdx* mouse model is not an adequate representation of the human disease. The only muscle that is consistently affected is the diaphragm, which shows membrane permeability and fibrotic replacement [5]. In addition, the* mdx* mice have a peak of pathology at 4 weeks old and otherwise the mice have very little pathology (reviewed in [6]).

Additional genetic engineering and breeding have been conducted to worsen the disease. A double knockout was generated with both dystrophin and its homolog utrophin deleted [7]. These mice are severely affected and only half live beyond 8 weeks old (Deconinck and [7]). Recently another knock-out combination of both dystrophin and telomerase also caused a very severe phenotype [8]. These two mouse models do not reflect the etiology found in humans as they require a second genetic mutation to attain a severe phenotype. Dystrophin mutation in humans is sufficient to induce a severe phenotype and, untreated, death before the end of the third decade. While these models' phenotypes are severe, the causes of the severity are different than those found in humans. These additional factors must then be compensated for during investigations bringing into question whether the research could be translated to patients.

The mouse model we are assessing is the genetically engineered *γ*-sarcoglycan (*Sgcg* ^−/−^) null mutation [9] on the DBA/2J (D2) background [10]. In the original publication, it was identified that the* Sgcg* ^−/−^ mice on the mixed C57BL/6J-129 background have a more severe cardiomyopathy than the* mdx* mice [9]. It was then demonstrated that the* Sgcg* ^−/−^ mutation is more severe when bred onto the D2 background [10]. The D2 mice carry a naturally occurring in-frame deletion within their Latent TGF*β* Binding Protein 4 (LTBP4) gene; the deletion segregates at a high level with severe disease [11]. The deletion causes a further increase in the already MD-elevated levels of active TGF*β*, which caused the excessive fibrosis observed in the* Sgcg* ^−/−^ D2 mice over three other comparison strains [11]. The D2 mouse strain also carries* Dyscalc*, a naturally occurring gene locus. This locus is linked to increased dystrophic cardiac calcinosis in numerous mouse lines including the D2, compared to mice lacking the* Dyscalc* gene locus [12]. Presence of the* Dyscalc* locus is sufficient to induce calcified lesions as a result of calcium deposits that accumulate after myofibers have necrosed, even in the wild type D2 mice. The causative gene is still under dispute, Abcc6 [13] or Emp3 [14]. While the products of these genes are known their exact functions are still under investigation. Additional breeding with the* mdx* mutation demonstrated that the* mdx* mutation presents more severely when bred onto the D2 background [15].

The ultimate usefulness of animal models is in the testing of potential patient therapies. Currently MD patients receive corticosteroids to diminish the skeletal deformities associated with MD and to keep the patients mobile as long as possible. Corticosteroids have not been proven useful in the animal models [16]; they have limited usefulness in humans as well. They are associated with significant side-effects [17, 18] and are not tolerated well for the long periods required for this chronic disease [17, 18]. Recently, it is also advised to prophylactically prescribe angiotensin receptor blockers or angiotensin converting enzyme inhibitors (reviewed in [19]). Angiotensin receptor blockers have shown efficacy in both the* mdx* mouse model [20] and in humans [21]. Furthermore, two of the most promising future therapies for a subset of MD patients are exon-skipping and read-through technologies [22, 23]. Both of these therapies, now on the brink of phase 2 and 3 trials, have proven efficacious in restoring dystrophin expression in the* mdx* mouse model [24]. These two therapies are examples from many more promising therapies justifying the further production and use of MD mouse models.

## 2. Materials and Methods

*Animals*. Mice were housed following UIC, national and international animal welfare protocols. A 12-hour light/dark cycle is kept year round within the animal facility; food and water are ad libitum. The mice are watched by the veterinary staff of the Biological Research Laboratory at UIC. Animals were housed 5 to a cage after weaning. DBA2/J (D2) mice with the* Sgcg* ^+/−^ were graciously provided by Dr. Elizabeth McNally. The mice have since been bred using Het (*Sgcg* ^+/−^) × Het breeding pairs and randomized selection of breeds to reduce user induced genetic drift. Het × Het and Het × KO (*Sgcg* ^−/−^) breeding pairs were established to provide the necessary animals for this study.* Mdx* mice were acquired from Jackson Laboratories (Bar Harbor Maine); they were housed in the animal facility to ensure identical environments.

*Histology*. Following harvest the tissues designated for paraffin imbedding was placed into 1.5 mL tubes filled with 1 mL of neutral buffered formalin. The fixed tissues were taken to the UIC RRC Histology Core for paraffin imbedding, slicing, Masons Trichrome, Pico Sirius Red, and H&E staining. The resulting slides were imaged on an Aperio ScanScope CS (Leica Biosystems, Nussloch Germany) whole slide imager. Image Scope (Leica Biosystems) was used to visualize the slide images for analysis. ImageJ was used to quantify fiber size variability.

*Evans Blue Dye (EBD)*. Two days before harvest each mouse is injected with EBD (Sigma Aldrich) at 5 *μ*L per gram of animal weight. EBD at 10 mg/mL in PBS was aliquoted and stored at −20C. Following sacrifice, tissue samples were minced and weighed in prelabelled 1.5 mL tubes. The tissues were frozen in liquid nitrogen and stored at −80C until processing. Tissues to be assayed were removed from the −80 freezer and 1 mL of Formamide (Sigma Aldrich) was added to each sample. The samples were then mixed by vortexing and incubating at 55°C for two hours. Following incubation the samples are mixed again by vortexing and spun down at 3 k rpm for 1 minute. 200 *μ*L of each sample and standards in triplicate (0 *μ*g, 0.625 *μ*g, 1.25 *μ*g, 2.5 *μ*g, 5 *μ*g, 10 *μ*g, and 20 *μ*g of EBD) were transferred to a corresponding well in a 96-well plate and then assessed on a Bio-Tek Synergy HT plate reader at 620 nm. The standards are used to create a linear fit equation, which is then used to calculate the ug EBD concentration of each sample. The *μ*g EBD concentration of each sample is then divided by the initial tissue weight giving a measure of *μ*g EBD/mg tissue weight for each sample. All samples are normalized to the kidney average to mitigate possible injection errors and equalize the dose between mice.

*Hydroxyproline Assay (HOP)*. The HOP protocol used follows a modified protocol from Flesch et al. [25]. Tissue samples were minced and weighed in prelabelled 1.5 mL tubes. 1 mL of 6 M hydrochloric acid (Sigma Aldrich, St. Louis, MO) was added to each tube. The tubes were heated at 105°C for 3 hours. After the heating period the samples were removed from the heat and left to cool to room temperature. 10 *μ*L of each sample was added to a clean, labelled, 1.5 mL tube with 150 *μ*L of isopropanol. After being mixed, 75 *μ*L of Solution A (1 part chloramine T (70 mg chloramine T (Sigma Aldrich) + 1 mL H_2_O) to 4 parts Acetate Citrate Buffer (57 g of Sodium Acetate (Sigma Aldrich), anhydrous, 435 mL of 1 M NaOH, 33.4 g of Citric Acid (Sigma Aldrich), 385 mL of Isopropanol, water to 1 liter)) is added. The samples are inverted twenty times and left to sit at room temperature for 10 minutes. Immediately following that, 1 mL of Solution B (3 parts Ehrlich's Reagent (3 g of p-dimethylaminobenzaldehyde (Sigma Aldrich), 10 mL of EtOH, 675 *μ*L of sulfuric acid (Thermo Fischer Scientific, Waltham MA) (mixed in drop by drop) to 13 parts isopropanol)) was added. The samples are again inverted twenty times to mix and then immediately placed into a 58°C water bath. Following a 30 minute incubation the samples are removed from the water bath, quickly mixed, and then buried in ice to quench the reaction. Cooled samples are then spun at 5 k for 1 minute, and 200 *μ*L of each sample is moved to a corresponding well in a 96-well plate. Along with standards in triplicate (0, 50, 100, 500, 100, 500, 1000, and 2000 mM Hydroxyproline (Sigma Aldrich)) the samples are assessed by a Bio-Tek Synergy HT (Bio-Tek, Winooski VT) plate reader at 558 nm. The standards are used to create a linear fit equation, which is then used to calculate the mM hydroxyproline concentration of each sample. The mM hydroxyproline concentration is then divided by the initial tissue weight, giving a final mM hydroxyproline/tissue weight (mg) concentration for each sample.

*Plethysmography*. Three days before harvest respiratory function was assessed for each mouse. The Buxco Small Animal Plethysmography (Buxco/DSI, St. Paul MN) set-up, using FinePointe (Buxco/DSI), was used. In brief, the machine is calibrated before each day's assessments. Mice are loaded into chambers individually, given an adjustment period, and then assessed for 15 minutes each. Each animal is assessed in at least two chambers sequentially. Breath frequency (*f*) was used to cull extraneous data sets from any instances in which the animal may have held its breath or breathed very rapidly. This was done by finding the average* f* for each mouse per total session. Standard deviation was calculated by Microsoft Excel and any data sets falling outside of 1 standard deviation were removed from that individual mouse's data set. The new average for each mouse was then calculated. Enhanced pause (Penh) is a mathematical comparison between early and late expirations a higher value represents increased pathology due to slower—more fibrotic—late expiration. It is calculated by FinePointe as Penh = (PEF/PIF) × (Te/Rt − 1), where PEF is peak expiratory height, PIF is peak inspiratory height, Te is expiratory time, and Rt is time to expire 65% of the volume.

*Echocardiography*. Cardiac function was assessed by Dr. Robert Gaffin of the UIC Center for Cardiovascular Research Physiology Core Labs using a Vevo 2100, with the manufacture's supplied software.

*Statistics*. Statistical analysis was performed using Student* t*-tests on Microsoft Excel.

## 3. Results

Multiple murine MD mouse lines have been utilized by researchers to investigate the etiology, pathology, and possible treatments for various forms of this devastating disease. In addition, various comparisons have also been conducted ([10, 15, 26] as examples) to identify the proper model for each experiment. We now present data comparing two mutations which model the most common form of MD that of MD generated by mutations within the dystrophin-glycoprotein proteins. We have time course comparisons of biochemical, histological, and functional characterizations for the* mdx* and* Sgcg* ^−/−^ D2 mouse strains.

### 3.1. Animal Weights

Animal weights are an initial test of MD disease severity. It is well known that mild MD is associated with an increase in animal mass. Alternatively, a more severe MD is associated with decreased animal mass, likely as a result of muscle atrophy [10, 27]. Others have reported that the* mdx* mice gain weight with respect to their littermate controls [27] indicating a mild disease course and presumably due to hypertrophy. We now show that the* Sgcg* ^−/−^ D2 mice trend to lower weights than their WT controls at 4 and 12 weeks (Figure 2). We also show these animal weights to aid in the comparative analysis of the functional assessments below.

### 3.2. Histologic Assessments

**Figure 3:**
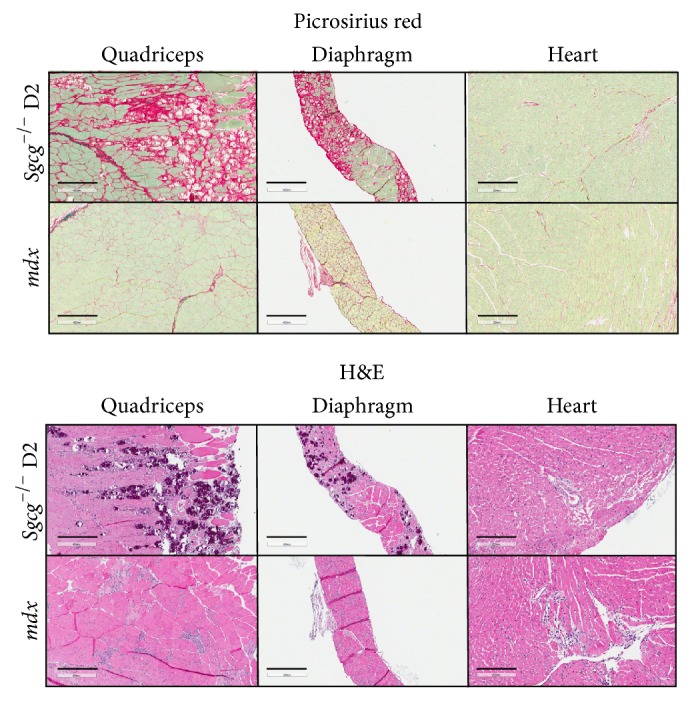
The* Sgcg* ^−/−^ D2 mice displayed the most severe pathology in histochemical assessments. By Pico Sirius Red staining the quadriceps and diaphragm of the* Sgcg* ^−/−^ D2 displayed far more red regions which correspond to fibrotic scarring than the* mdx* tissues. The hematoxylin and eosin pictures demonstrated the same fibrosis. The fibrosis in the cardiac left ventricles appeared quite similar between the two genotypes. Both mouse models demonstrated interstitial and perivascular fibrosis. Quadriceps and diaphragm legend bars represent 400 *μ*m. Heart legend bars represent 200 *μ*m.

The* Sgcg* ^−/−^ D2 quadriceps muscles have increased fibrosis, increased variation in fiber size, and increased central nuclei compared to age matched* mdx* mice (all 12 weeks old, Figure 3). In quadriceps the Pico Sirius Red staining indicates increased red, fibrotic areas in the* Sgcg* ^−/−^ D2 tissues (Figure 3). Similarly, the representative hematoxylin and eosin (H&E) images demonstrate larger blue/purple, fibrotic regions in the* Sgcg* ^−/−^ D2 mice in quadriceps. Quadriceps muscles from two* mdx* and two* Sgcg* ^−/−^ D2 mice were quantified for fiber size variability (FSV) with ImageJ software. As only two quadriceps muscles were compared, statistics were not performed. The* Sgcg* ^−/−^ D2 mice had a wider variation in fiber size (*Sgcg* ^−/−^ D2; 3453 ± 2023 mm and* mdx*; 2470 ± 1830) indicating more ongoing regeneration and a more severe phenotype.

As expected the diaphragms from both mouse groups contained predominant interstitial fibrosis (central panels, Figure 3) and were several cell layers thicker than wild type (wild type images in [9, 10]). The* Sgcg* ^−/−^ D2 diaphragms contained the most fibrotic areas by Pico Sirius Red staining and the largest accumulation of blue/purple regions in the H&E stained sections.

The cardiac ventricles appeared similarly affected by the muscular dystrophy pathology. Both images demonstrate interstitial and perivascular fibrosis. In addition the* mdx* heart contains some myofibroblast replacement of myofibers in the central portion of the H&E image.

### 3.3. Membrane Permeability

**Figure 4:**
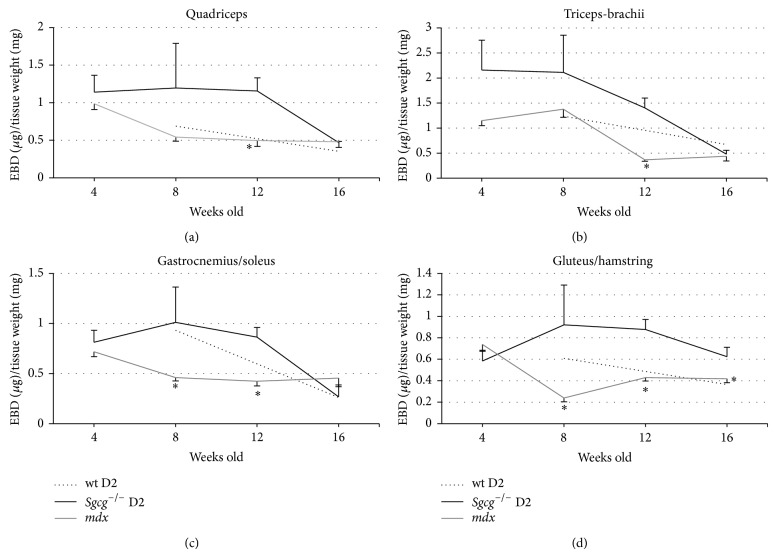
The* Sgcg* ^−/−^ D2 muscles demonstrated significantly increased membrane permeability at 12 weeks old. (a)* Sgcg* ^−/−^ D2 mice showed statistically higher EBD in their Quadriceps at 12 weeks compared to the* mdx* mice. (b)* Sgcg* ^−/−^ D2 mice showed statistically higher EBD in their Triceps-Brachii at 12 weeks compared to the* mdx* mice. (c)* Sgcg* ^−/−^ D2 showed statistically higher EBD in their Gastrocnemius and Soleus at 8 and 12 weeks compared to the* mdx* mice. (d)* Sgcg* ^−/−^ D2 Gluteus and Hamstrings showed higher EBD than the* mdx* mice at 8, 12, and 16 weeks. ^∗^ *P* < 0.05 significance versus* Sgcg* ^−/−^ D2 animals.* Sgcg* ^+/+^ D2 at 4, 12 weeks *n* = 0, all others *n* = 3–15.

We assessed membrane permeability by quantitatively measuring the amount of Evans blue dye (EBD) that entered the damaged muscle fibers [10]. EBD binds albumin and only enters damaged muscle fibers [28]. The* Sgcg* ^−/−^ D2 skeletal muscle had significantly higher membrane permeability at 12 weeks across all four tissues assessed. Additionally the* Sgcg* ^−/−^ D2 mice had greater membrane permeability at 8 weeks in the Gastrocnemius/Soleus and at 8 and 16 weeks in the Gluteus/Hamstrings. Interestingly at 16 weeks an improvement in membrane permeability was identified in all* Sgcg* ^−/−^ D2 skeletal muscles assessed (Figure 4). The* mdx* disease was most severe at the 4-week time point which was also followed by an improvement (Figure 4). This result is consistent with published reports that the* mdx* limb based skeletal muscles are severely affected at 4 weeks old and then improve [27, 29]. This indicates that the* Sgcg* ^−/−^ D2 mice succumb more severely to early disease pathology than the* mdx* mice and that both recover slightly due to as yet unknown reasons.

### 3.4. Collagen Content

**Figure 5:**
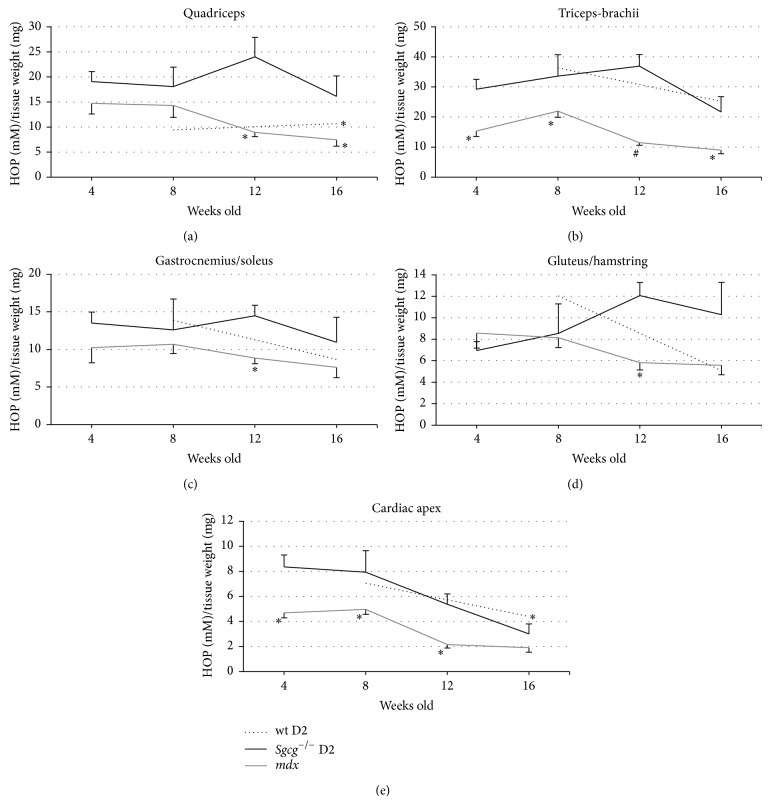
The* Sgcg* ^−/−^ D2 muscles were consistently more fibrotic than the age matched* mdx* mice by hydroxyproline assay.* Sgcg* ^−/−^ D2 mice showed significantly higher levels of Quadriceps fibrosis at 12 and 16 weeks versus the* mdx* mice and significantly more fibrosis than their* Sgcg* ^+/+^ D2 controls at 16 weeks.* Sgcg* ^−/−^ D2 mice had significantly higher fibrosis content in their Triceps-Brachii at all-time points compared to the* mdx* mice. At 12 weeks* Sgcg* ^−/−^ D2 mice have higher fibrosis content than the* mdx* mice in their Gastrocnemius/Soleus muscles. A similar pattern was seen in the Gluteus/Hamstring muscle group, and the* Sgcg* ^−/−^ mice had significantly higher fibrosis content at 12 weeks than the* mdx* mice.* Sgcg* ^−/−^ mice showed greater collagen content at 4, 8, and 12 weeks compared to the* mdx* mice. Additionally the* Sgcg* ^+/+^ mice had higher collagen content than the* Sgcg* ^−/−^ mice at 16 weeks. ^∗^ *P* < 0.05, ^#^ *P* < 0.001 significance versus* Sgcg* ^−/−^ D2 animals.* Sgcg* ^+/+^ D2 at 4 and 12 weeks *n* = 0;* Sgcg* ^+/+^ D2 at 8 weeks *n* = 2; all others *n* = 3–16.

To assess fibrosis we performed a quantitative hydroxyproline assay on the harvested muscles [10]. Hydroxyproline is a modified amino acid, which is only present in collagen and therefore is a quantitative marker for intramuscular fibrosis. The* Sgcg* ^−/−^ D2 muscles contained the largest amount of hydroxyproline in all limb-based muscles tested at almost all ages (Figure 5). This increase was significant in quadriceps at 12 and 16 weeks, in triceps-brachii at all times tested, Gastrocnemius/Soleus at 12 weeks, and Gluteus/Hamstring at 12 weeks. The only point when the* mdx* tissue contained more hydroxyproline was at 4 weeks in the gluteus/hamstring. Once again, the phenotype of the* Sgcg* ^−/−^ D2 mice progressively worsened until 12 weeks of age and then improved, while the* mdx* mouse phenotype improved after 4 weeks of age.

WT D2 mice displayed greater fibrosis at various time points and across all tissues compared to the* mdx* mouse. The D2 mouse line contains an in-frame deletion within the LTBP-4 gene. This mutation induces greater TGF-*β*1 activity leading to increased fibrosis in the D2 mouse line [11]. Fukada et al. 2009 do show that the* mdx* mutation bred into the D2 line is more severe than the mutation in the C57BL/10 line; however the assessment ages are drastically different than those being used in the current study. Therefore, we cannot compare the observed decrease in fibrosis in our mice with the continued worsening phenotype observed by Fukada et al. [15].

The fibrosis content of the diaphragm was considered separately. The diaphragm develops differently than the limb-based skeletal muscles [30], is innervated differently [30], contains roughly the same quantity of Pax7 positive cells as the EDL and Biceps [31], and is the most severely affected muscle in the* mdx* [5] and* Sgcg* ^−/−^ D2 mice [10, Figure 3]. Many have conjectured as to why the diaphragm is the most severely affected muscle [5], but a well-accepted hypothesis has not been developed. Comparing the* mdx* and* Sgcg* ^−/−^ D2 diaphragm fibrosis revealed no significant differences.

The cardiac ventricle fibrosis also displayed a different pattern than the limb based skeletal muscles (Figure 5(e)).* Mdx* mice had less cardiac fibrosis than the* Sgcg* ^−/−^ D2 animals at all ages and this reached significance at 4, 8, and 12 weeks. Interestingly, by 16 weeks of age the HOP values showed no significant differences. Furthermore, hearts from both animal groups demonstrated reduced fibrosis after 8 weeks of age.

### 3.5. Plethysmography

We also analyzed the two different MD mouse models for respiratory function. Plethysmography is an often used and reliable assessment technique of diaphragm function [32, 33]. The diaphragm is normally very thin and very pliable. In muscular dystrophy the diaphragm becomes thick and rigid due to scar tissue [10]. Once rigid, the lungs require extended time for inspiration and expiration and a longer pause is apparent on the breath wave [32, 33]. These three metrics (times of inspiration (Ti), expiration (Te), and pause (PenH)) all measure the elasticity of the diaphragm and to a lesser extent the strength of the intercostal muscles.

**Figure 6:**
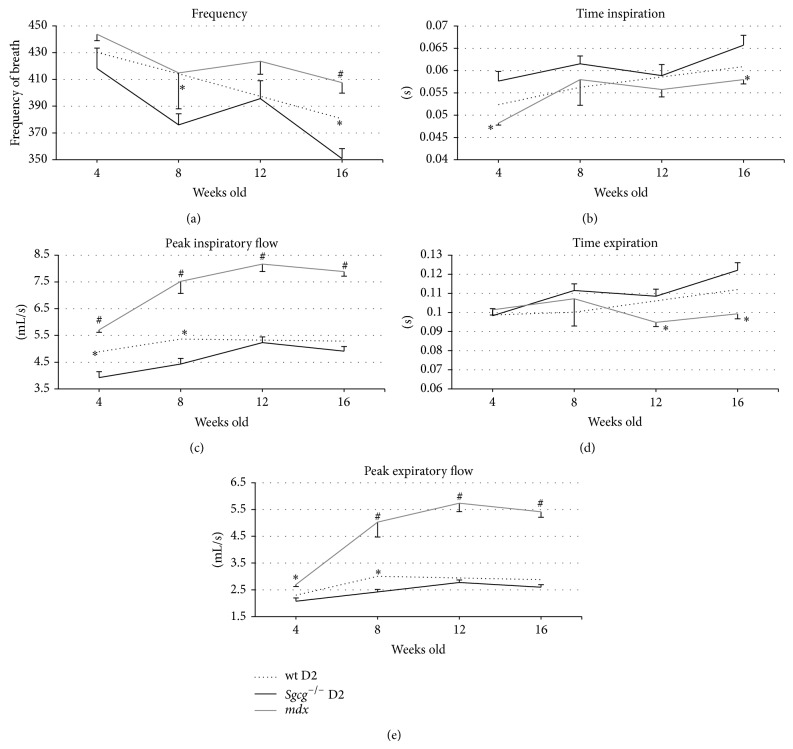
The* Sgcg* ^−/−^ D2 mice consistently had a more severe respiratory phenotype by plethysmography then the* mdx* animals. (a) At 8 and 16 weeks the* Sgcg* ^−/−^ D2 mice breathed significantly slower than the* Sgcg* ^+/+^ D2 mice. At 16 weeks the* Sgcg* ^−/−^ D2 mice also breathed significantly slower than the* mdx* mice. (b)* Sgcg* ^−/−^ D2 mice inhaled (Ti) slower than the* mdx* mice at 4 and 16 weeks. (c)* Sgcg* ^−/−^ D2 mice had a lower peak inspiratory flow (PIFb) than the* mdx* mice at all-time points and lower than the* Sgcg* ^+/+^ D2 mice at 4 and 8 weeks. (d)* Sgcg* ^−/−^ D2 mice exhaled slower than the* mdx* mice at 12 and 16 weeks. (e)* Sgcg* ^−/−^ D2 mice had a lower peak expiratory flow (PEFb) than the* mdx* mice at all-time points and lower flow than the* Sgcg* ^+/+^ D2 at 8 weeks. ^∗^ *P* < 0.05, ^#^ *P* < 0.001 significance versus* Sgcg* ^−/−^ D2 animals.* Sgcg* ^+/+^ D2 at 12 weeks *n* = 0, all others *n* = 4–10.

*Sgcg* ^−/−^ D2 animals had significantly longer time inspiration (Ti) and time expiration (Te) than the* mdx* mice at 8, 12, and 16 and 12 and 16 weeks, respectively. Ti and Te are the measure of time that passes while the animal inhales or exhales, respectively. Additionally the* Sgcg* ^−/−^ D2 animals had significantly slower peak flows (fastest movement of air into the lungs) for inspiration and expiration (Figures 6(c) and 6(e)). Consistent with the longer inspiration and expiration parameters the* Sgcg* ^−/−^ D2 mice had significantly reduced breath frequency (breathes per minute, Figure 6(a)). The remainder of the plethysmography data can be accessed online in Supplemental Figure 1 in Supplementary Material available online at http://dx.doi.org/10.1155/2015/131436; when evaluating some of these parameters please be aware that the* mdx* mice are significantly larger than the D2 mice (Figure 1) and that this will affect many of the volume calculations. No difference was found in the Penh value between the* mdx* and* Sgcg* ^−/−^ D2 mice.

The D2 animals, regardless of genotype, were found to have reduced Ti, Te, Peak flow inspiration, and expiration at numerous time points compared to the* mdx* animals. Presently it can be hypothesized that the LTBP-4 mutations could be responsible for this impaired respiratory function due to increased fibrosis.

Our data is consistent with previously published plethysmography data. The following parameters, frequency, PIF, and PEF, were shown to be similar to the numbers presented in the current paper for 12-week old* mdx* mice [32]. These parameters were all statistically different from the wild type C57BL/10 mice used as controls in that experiment. The* mdx* animals also had pathologically lower frequency of breaths and decreased peak flows than the C57BL/10 animals.

### 3.6. Echocardiography

**Figure 7:**
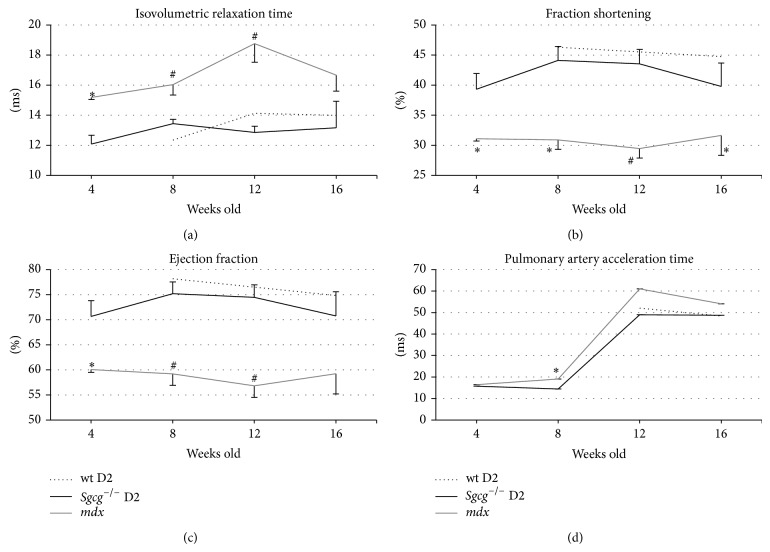
The* mdx* mice demonstrated a more severe cardiac functional pathology. (a)* Sgcg* ^−/−^ D2 mice had a shorter isovolumetric relaxation time (IVRT) than the* mdx* mice at all-time points. (b) The* Sgcg* ^−/−^ mice have a higher fractional shortening (FS%). (c)* Sgcg* ^−/−^ have a greater ejection fraction (EF%) than the* mdx* mice at 4, 8, and 12 weeks. (d) Pulmonary artery acceleration time (PA AT) was lower in the* Sgcg* ^−/−^ mice at 8 weeks compared to the* mdx* mice. ^∗^ *P* < 0.05, ^#^ *P* < 0.001 significance versus* Sgcg* ^−/−^ D2 animals.* Sgcg* ^+/+^ D2 at 4 weeks *n* = 0, all others *n* = 4–23.

To further assess the functional effects of MD on these two mouse strains an echocardiography time-course was conducted. Despite demonstrating increased ventricular fibrosis compared to the* mdx* mice (Figure 5(e)), the* Sgcg* ^−/−^ D2 animal's cardiac function was preserved at wild type levels (Figure 7). The* mdx* animals had significantly higher isovolumetric relaxation time (IVRT) and smaller fractional shortening and ejection fraction. The* mdx* animals had significantly higher isovolumetric relaxation time (IVRT), indicative of diastolic dysfunction, and smaller fractional shortening and ejection fraction, indicative of systolic dysfunction. Pulmonary artery acceleration time (PAAT) is a surrogate measure of pulmonary hypertension. A decrease in PAAT indicates increased pulmonary hypertension. PAAT was lower in the* Sgcg* ^−/−^ D2 animals than the* mdx* and significantly lower at 8 weeks of age, indicating that the* Sgcg* ^−/−^ D2 animals were more severely affected than the* mdx* mice. Please see supplemental Figure 2 for complete echocardiography data, and the authors would like to urge caution when comparing values that are dependent upon mouse size.

Despite increased fibrosis in the* Sgcg* ^−/−^ D2 hearts (Figures 3 and 5(e)) and previous publications [9], the* mdx* mice demonstrated significantly more severe cardiac pathology by echocardiography. Currently we do not have a full explanation for this, although it is obvious that fibrosis is only one of many pathologic occurrences in a muscular dystrophic heart. Conduction system defects have been identified in the* mdx* mouse heart [34]. In addition calcium is mislocalized in dystrophy [35] and therefore excitation and contraction will be detrimentally affected.

## 4. Conclusion

Animal models are an essential part of investigating disease etiology, progression, and preclinical trials. As muscular dystrophy (MD) is the most prevalent human genetic disease these mouse models are essential and a clear understanding of their pathology is required, including comparisons between those models already available. Multiple mouse MD model comparisons have been conducted. Fukada et al. previously bred the* mdx* mutation into the D2 mouse strain and compared the resulting strain to the historical* mdx* (C57BL/10) mouse [15]. The* mdx *mutation was identified to be more severe on the D2 background by muscle weights, fewer myofibers, increased fibrosis, and decreased strength. These authors identified that the D2 mouse strain has decreased satellite cell self-renewal and reasoned that this was the pertinent phenotypic causing difference [15]. An additional pair of manuscripts phenotypically described the* Sgcg* ^−/−^ mutation in four commonly used strains [10]. The D2 mice were found to have the most severe disease and using GWAS this was found to be largely due to a deletion within the Latent TGF*β* Binding Protein 4 [11]. We now wished to directly compare the severe* Sgcg* ^−/−^ D2 mouse to the highly used* mdx* mouse histologically, biochemically, and functionally.

The* Sgcg* ^−/−^ D2 mice were found to have increased membrane permeability and fibrosis in the limb based skeletal muscles analyzed. Many of the* Sgcg* ^−/−^ D2 muscles demonstrated statistically increased pathology for these two characteristics at most time points analyzed after the initial 4 week time point. The histology confirmed a more severe disease in the* Sgcg* ^−/−^ D2 mice at the 12-week time point. Importantly the* Sgcg* ^−/−^ D2 mice demonstrated increased pulmonary hypertension by echocardiography compared to the* mdx* mice at all ages. Furthermore, by histology the* Sgcg* ^−/−^ D2 mouse also displayed more severe pathology in the quadriceps and diaphragm than the* mdx* model. In addition, as has often been noted, the diaphragms from both groups of mice are the most severely affected skeletal tissues. The reason behind the robust diaphragm pathology still eludes scientists.

Many of the phenotypic differences are trending to significance. One limitation of these experiments is the limited number of animals used, due to the time-course nature of the data. Muscular dystrophy in mice and humans is notoriously variable even within the same organism (mice [10] and humans [36, 37]). Despite this caveat the trends are upheld for many tissues and across many time points indicating that the conclusions drawn are sound and the* Sgcg* ^−/−^ D2 mice present a better model of the human disease.

Both the* mdx* and* Sgcg* ^−/−^ D2 mouse phenotypes improve after an initial bout of pathology. Although this has been identified and discussed in the* mdx* mice for many years [5] we have not found an explanation for the reduction. In the current experiments the* mdx* mouse pathology is most severe at the 4 week time point, while the* Sgcg* ^−/−^ mice are most severely affected at 12 weeks. Possible mechanisms behind this phenotype recovery are; satellite cell recovery, immune response is dampened, beneficial scar tissue remodeling, reduced movement or, likely a combination of these and other mechanisms. Additional important questions remain. It would be of great interest to know why the different mouse strains have their peak pathologies at different ages. And it would be ideal to understand if this recovery mechanism can be harnessed for human patients.

Even though we argue that the* Sgcg* ^−/−^ D2 mouse is a better model of human muscular dystrophy we must remember that it is still a model. There are many considerations when using mouse models to represent human disease (reviewed in [38]). There are the obvious differences: bipedal versus quadripedal, free living with multiple stresses versus caged generally without physical activity, age, and size. The less obvious differences must also be considered. Slight differences in gene expression patterns, in signaling pathways, and in immune responses exist. As an example, important interspecies differences in the Notch signaling pathway have been identified [39].

To summarize, in this paper we provide data supporting that the* Sgcg* ^−/−^ D2 mouse is a more appropriate mouse model for muscular dystrophy research. While the mutations responsible for the development of muscular dystrophy within the strains are different, we set out to investigate which model develops a more severe—more human—phenotype. Gathered data demonstrates that the* Sgcg* ^−/−^ D2 mouse develops a more severe phenotype in most assessed variables with the exception of cardiac function.

## Supplementary Material

Supplementary material encompasses the overall data from the plethysmography and echocardiography assessments taken during experiments. D2 Sgcg +/+ are WT, D2 Sgcg -/- are KO, Mdx are mdx. Mouse count is listed as well as the average and standard error of the mean (SEM) for each mouse group.
