# Supplementary material for: Biochemical and Functional Comparisons of mdx and Sgcg −/− Muscular Dystrophy Mouse Models
Source: Biomed Res Int. 2015 May 3;2015:131436. doi: 10.1155/2015/131436 (PMC4433636; doi:10.1155/2015/131436)
Supplement: Supplementary file 1 — Supplementary material encompasses the overall data from the plethysmography and echocardiography assessments taken during experiments. D2 Sgcg +/+ are WT, D2 Sgcg -/- are KO, Mdx are mdx. Mouse count is listed as well as the average and standard error of the mean (SEM) for each mouse group. [file 131436.f1.pptx]

## Slide 1
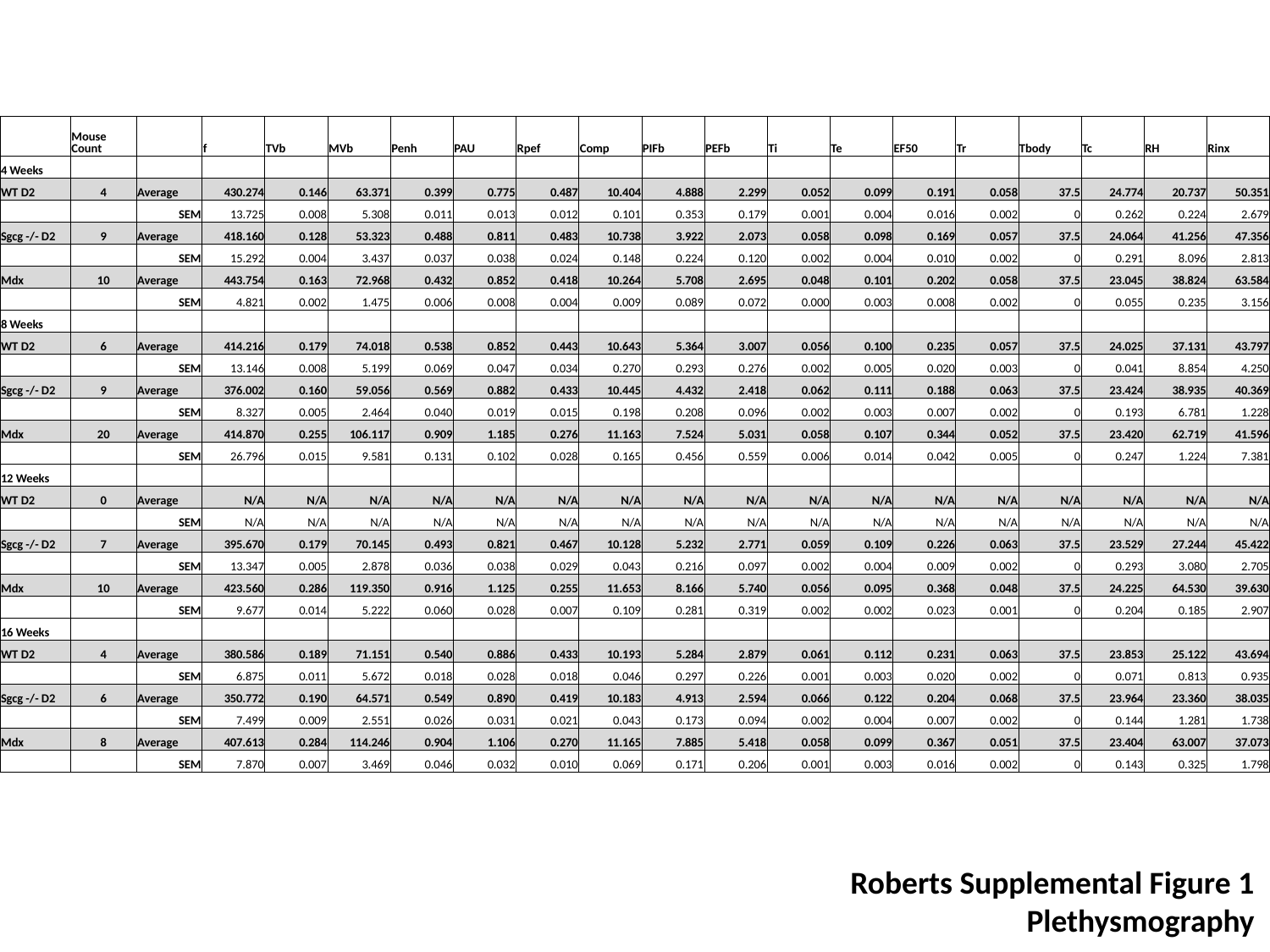

| | Mouse Count | | f | TVb | MVb | Penh | PAU | Rpef | Comp | PIFb | PEFb | Ti | Te | EF50 | Tr | Tbody | Tc | RH | Rinx |
| --- | --- | --- | --- | --- | --- | --- | --- | --- | --- | --- | --- | --- | --- | --- | --- | --- | --- | --- | --- |
| 4 Weeks | | | | | | | | | | | | | | | | | | | |
| WT D2 | 4 | Average | 430.274 | 0.146 | 63.371 | 0.399 | 0.775 | 0.487 | 10.404 | 4.888 | 2.299 | 0.052 | 0.099 | 0.191 | 0.058 | 37.5 | 24.774 | 20.737 | 50.351 |
| | | SEM | 13.725 | 0.008 | 5.308 | 0.011 | 0.013 | 0.012 | 0.101 | 0.353 | 0.179 | 0.001 | 0.004 | 0.016 | 0.002 | 0 | 0.262 | 0.224 | 2.679 |
| Sgcg -/- D2 | 9 | Average | 418.160 | 0.128 | 53.323 | 0.488 | 0.811 | 0.483 | 10.738 | 3.922 | 2.073 | 0.058 | 0.098 | 0.169 | 0.057 | 37.5 | 24.064 | 41.256 | 47.356 |
| | | SEM | 15.292 | 0.004 | 3.437 | 0.037 | 0.038 | 0.024 | 0.148 | 0.224 | 0.120 | 0.002 | 0.004 | 0.010 | 0.002 | 0 | 0.291 | 8.096 | 2.813 |
| Mdx | 10 | Average | 443.754 | 0.163 | 72.968 | 0.432 | 0.852 | 0.418 | 10.264 | 5.708 | 2.695 | 0.048 | 0.101 | 0.202 | 0.058 | 37.5 | 23.045 | 38.824 | 63.584 |
| | | SEM | 4.821 | 0.002 | 1.475 | 0.006 | 0.008 | 0.004 | 0.009 | 0.089 | 0.072 | 0.000 | 0.003 | 0.008 | 0.002 | 0 | 0.055 | 0.235 | 3.156 |
| 8 Weeks | | | | | | | | | | | | | | | | | | | |
| WT D2 | 6 | Average | 414.216 | 0.179 | 74.018 | 0.538 | 0.852 | 0.443 | 10.643 | 5.364 | 3.007 | 0.056 | 0.100 | 0.235 | 0.057 | 37.5 | 24.025 | 37.131 | 43.797 |
| | | SEM | 13.146 | 0.008 | 5.199 | 0.069 | 0.047 | 0.034 | 0.270 | 0.293 | 0.276 | 0.002 | 0.005 | 0.020 | 0.003 | 0 | 0.041 | 8.854 | 4.250 |
| Sgcg -/- D2 | 9 | Average | 376.002 | 0.160 | 59.056 | 0.569 | 0.882 | 0.433 | 10.445 | 4.432 | 2.418 | 0.062 | 0.111 | 0.188 | 0.063 | 37.5 | 23.424 | 38.935 | 40.369 |
| | | SEM | 8.327 | 0.005 | 2.464 | 0.040 | 0.019 | 0.015 | 0.198 | 0.208 | 0.096 | 0.002 | 0.003 | 0.007 | 0.002 | 0 | 0.193 | 6.781 | 1.228 |
| Mdx | 20 | Average | 414.870 | 0.255 | 106.117 | 0.909 | 1.185 | 0.276 | 11.163 | 7.524 | 5.031 | 0.058 | 0.107 | 0.344 | 0.052 | 37.5 | 23.420 | 62.719 | 41.596 |
| | | SEM | 26.796 | 0.015 | 9.581 | 0.131 | 0.102 | 0.028 | 0.165 | 0.456 | 0.559 | 0.006 | 0.014 | 0.042 | 0.005 | 0 | 0.247 | 1.224 | 7.381 |
| 12 Weeks | | | | | | | | | | | | | | | | | | | |
| WT D2 | 0 | Average | N/A | N/A | N/A | N/A | N/A | N/A | N/A | N/A | N/A | N/A | N/A | N/A | N/A | N/A | N/A | N/A | N/A |
| | | SEM | N/A | N/A | N/A | N/A | N/A | N/A | N/A | N/A | N/A | N/A | N/A | N/A | N/A | N/A | N/A | N/A | N/A |
| Sgcg -/- D2 | 7 | Average | 395.670 | 0.179 | 70.145 | 0.493 | 0.821 | 0.467 | 10.128 | 5.232 | 2.771 | 0.059 | 0.109 | 0.226 | 0.063 | 37.5 | 23.529 | 27.244 | 45.422 |
| | | SEM | 13.347 | 0.005 | 2.878 | 0.036 | 0.038 | 0.029 | 0.043 | 0.216 | 0.097 | 0.002 | 0.004 | 0.009 | 0.002 | 0 | 0.293 | 3.080 | 2.705 |
| Mdx | 10 | Average | 423.560 | 0.286 | 119.350 | 0.916 | 1.125 | 0.255 | 11.653 | 8.166 | 5.740 | 0.056 | 0.095 | 0.368 | 0.048 | 37.5 | 24.225 | 64.530 | 39.630 |
| | | SEM | 9.677 | 0.014 | 5.222 | 0.060 | 0.028 | 0.007 | 0.109 | 0.281 | 0.319 | 0.002 | 0.002 | 0.023 | 0.001 | 0 | 0.204 | 0.185 | 2.907 |
| 16 Weeks | | | | | | | | | | | | | | | | | | | |
| WT D2 | 4 | Average | 380.586 | 0.189 | 71.151 | 0.540 | 0.886 | 0.433 | 10.193 | 5.284 | 2.879 | 0.061 | 0.112 | 0.231 | 0.063 | 37.5 | 23.853 | 25.122 | 43.694 |
| | | SEM | 6.875 | 0.011 | 5.672 | 0.018 | 0.028 | 0.018 | 0.046 | 0.297 | 0.226 | 0.001 | 0.003 | 0.020 | 0.002 | 0 | 0.071 | 0.813 | 0.935 |
| Sgcg -/- D2 | 6 | Average | 350.772 | 0.190 | 64.571 | 0.549 | 0.890 | 0.419 | 10.183 | 4.913 | 2.594 | 0.066 | 0.122 | 0.204 | 0.068 | 37.5 | 23.964 | 23.360 | 38.035 |
| | | SEM | 7.499 | 0.009 | 2.551 | 0.026 | 0.031 | 0.021 | 0.043 | 0.173 | 0.094 | 0.002 | 0.004 | 0.007 | 0.002 | 0 | 0.144 | 1.281 | 1.738 |
| Mdx | 8 | Average | 407.613 | 0.284 | 114.246 | 0.904 | 1.106 | 0.270 | 11.165 | 7.885 | 5.418 | 0.058 | 0.099 | 0.367 | 0.051 | 37.5 | 23.404 | 63.007 | 37.073 |
| | | SEM | 7.870 | 0.007 | 3.469 | 0.046 | 0.032 | 0.010 | 0.069 | 0.171 | 0.206 | 0.001 | 0.003 | 0.016 | 0.002 | 0 | 0.143 | 0.325 | 1.798 |
Roberts Supplemental Figure 1
Plethysmography

## Slide 2
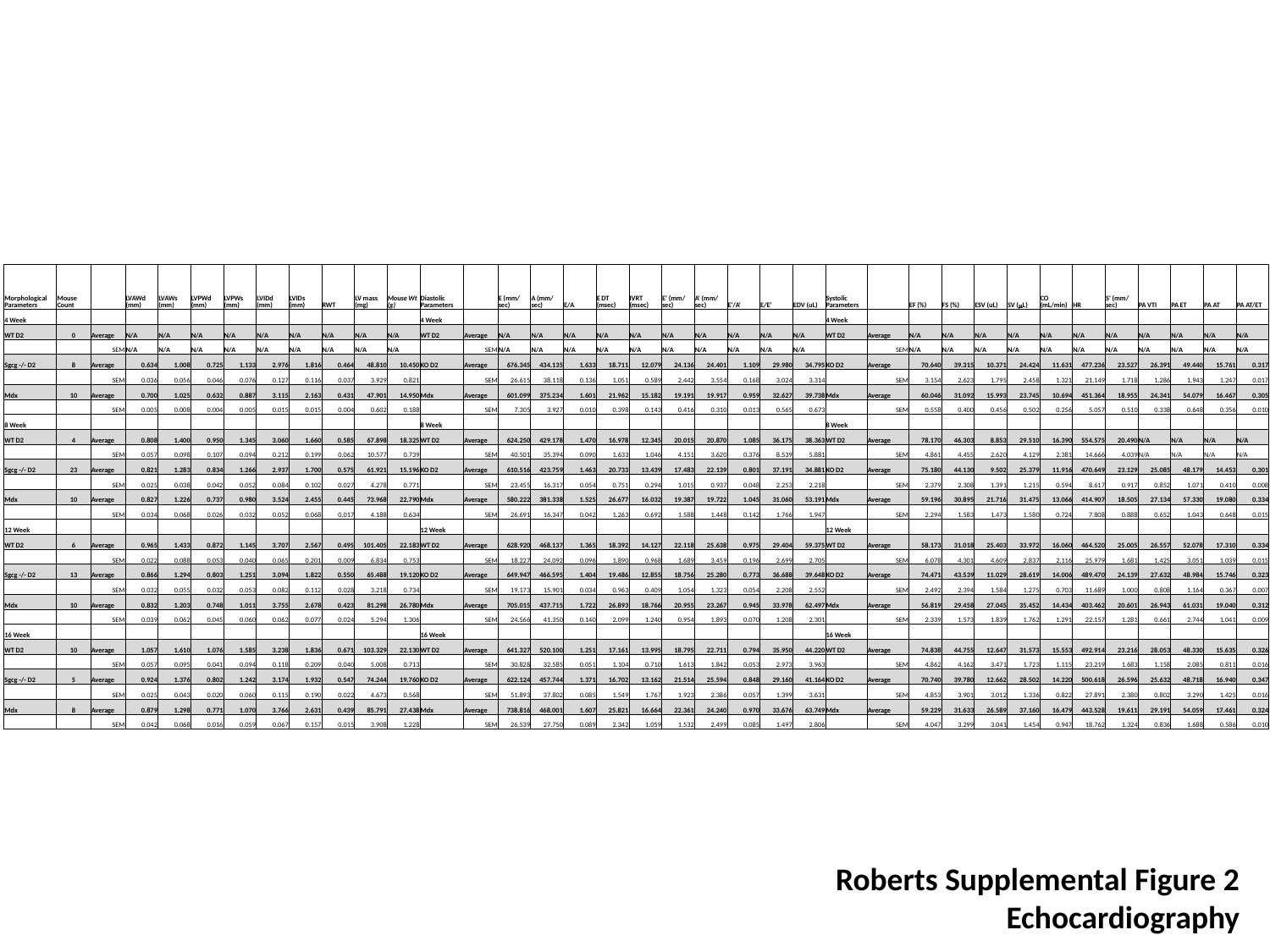

| Morphological Parameters | Mouse Count | | LVAWd (mm) | LVAWs (mm) | LVPWd (mm) | LVPWs (mm) | LVIDd (mm) | LVIDs (mm) | RWT | LV mass (mg) | Mouse Wt (g) | Diastolic Parameters | | E (mm/ sec) | A (mm/ sec) | E/A | E DT (msec) | IVRT (msec) | E' (mm/ sec) | A' (mm/ sec) | E'/A' | E/E' | EDV (uL) | Systolic Parameters | | EF (%) | FS (%) | ESV (uL) | SV (mL) | CO (mL/min) | HR | S' (mm/ sec) | PA VTI | PA ET | PA AT | PA AT/ET |
| --- | --- | --- | --- | --- | --- | --- | --- | --- | --- | --- | --- | --- | --- | --- | --- | --- | --- | --- | --- | --- | --- | --- | --- | --- | --- | --- | --- | --- | --- | --- | --- | --- | --- | --- | --- | --- |
| 4 Week | | | | | | | | | | | | 4 Week | | | | | | | | | | | | 4 Week | | | | | | | | | | | | |
| WT D2 | 0 | Average | N/A | N/A | N/A | N/A | N/A | N/A | N/A | N/A | N/A | WT D2 | Average | N/A | N/A | N/A | N/A | N/A | N/A | N/A | N/A | N/A | N/A | WT D2 | Average | N/A | N/A | N/A | N/A | N/A | N/A | N/A | N/A | N/A | N/A | N/A |
| | | SEM | N/A | N/A | N/A | N/A | N/A | N/A | N/A | N/A | N/A | | SEM | N/A | N/A | N/A | N/A | N/A | N/A | N/A | N/A | N/A | N/A | | SEM | N/A | N/A | N/A | N/A | N/A | N/A | N/A | N/A | N/A | N/A | N/A |
| Sgcg -/- D2 | 8 | Average | 0.634 | 1.008 | 0.725 | 1.133 | 2.976 | 1.816 | 0.464 | 48.810 | 10.450 | KO D2 | Average | 676.345 | 434.135 | 1.633 | 18.711 | 12.079 | 24.136 | 24.401 | 1.109 | 29.980 | 34.795 | KO D2 | Average | 70.640 | 39.315 | 10.371 | 24.424 | 11.631 | 477.236 | 23.527 | 26.391 | 49.440 | 15.761 | 0.317 |
| | | SEM | 0.036 | 0.056 | 0.046 | 0.076 | 0.127 | 0.116 | 0.037 | 3.929 | 0.821 | | SEM | 26.615 | 38.118 | 0.136 | 1.051 | 0.589 | 2.442 | 3.554 | 0.168 | 3.024 | 3.314 | | SEM | 3.154 | 2.623 | 1.795 | 2.458 | 1.321 | 21.149 | 1.718 | 1.286 | 1.943 | 1.247 | 0.017 |
| Mdx | 10 | Average | 0.700 | 1.025 | 0.632 | 0.887 | 3.115 | 2.163 | 0.431 | 47.901 | 14.950 | Mdx | Average | 601.099 | 375.234 | 1.601 | 21.962 | 15.182 | 19.191 | 19.917 | 0.959 | 32.627 | 39.738 | Mdx | Average | 60.046 | 31.092 | 15.993 | 23.745 | 10.694 | 451.364 | 18.955 | 24.341 | 54.079 | 16.467 | 0.305 |
| | | SEM | 0.005 | 0.008 | 0.004 | 0.005 | 0.015 | 0.015 | 0.004 | 0.602 | 0.188 | | SEM | 7.305 | 3.927 | 0.010 | 0.398 | 0.143 | 0.416 | 0.310 | 0.013 | 0.565 | 0.673 | | SEM | 0.558 | 0.400 | 0.456 | 0.502 | 0.256 | 5.057 | 0.510 | 0.338 | 0.648 | 0.356 | 0.010 |
| 8 Week | | | | | | | | | | | | 8 Week | | | | | | | | | | | | 8 Week | | | | | | | | | | | | |
| WT D2 | 4 | Average | 0.808 | 1.400 | 0.950 | 1.345 | 3.060 | 1.660 | 0.585 | 67.898 | 18.325 | WT D2 | Average | 624.250 | 429.178 | 1.470 | 16.978 | 12.345 | 20.015 | 20.870 | 1.085 | 36.175 | 38.363 | WT D2 | Average | 78.170 | 46.303 | 8.853 | 29.510 | 16.390 | 554.575 | 20.490 | N/A | N/A | N/A | N/A |
| | | SEM | 0.057 | 0.098 | 0.107 | 0.094 | 0.212 | 0.199 | 0.062 | 10.577 | 0.739 | | SEM | 40.501 | 35.394 | 0.090 | 1.633 | 1.046 | 4.151 | 3.620 | 0.376 | 8.539 | 5.881 | | SEM | 4.861 | 4.455 | 2.620 | 4.129 | 2.381 | 14.666 | 4.039 | N/A | N/A | N/A | N/A |
| Sgcg -/- D2 | 23 | Average | 0.821 | 1.283 | 0.834 | 1.266 | 2.937 | 1.700 | 0.575 | 61.921 | 15.196 | KO D2 | Average | 610.516 | 423.759 | 1.463 | 20.733 | 13.439 | 17.483 | 22.139 | 0.801 | 37.191 | 34.881 | KO D2 | Average | 75.180 | 44.130 | 9.502 | 25.379 | 11.916 | 470.649 | 23.129 | 25.085 | 48.179 | 14.453 | 0.301 |
| | | SEM | 0.025 | 0.038 | 0.042 | 0.052 | 0.084 | 0.102 | 0.027 | 4.278 | 0.771 | | SEM | 23.455 | 16.317 | 0.054 | 0.751 | 0.294 | 1.015 | 0.937 | 0.048 | 2.253 | 2.218 | | SEM | 2.379 | 2.308 | 1.391 | 1.215 | 0.594 | 8.617 | 0.917 | 0.852 | 1.071 | 0.410 | 0.008 |
| Mdx | 10 | Average | 0.827 | 1.226 | 0.737 | 0.980 | 3.524 | 2.455 | 0.445 | 73.968 | 22.790 | Mdx | Average | 580.222 | 381.338 | 1.525 | 26.677 | 16.032 | 19.387 | 19.722 | 1.045 | 31.060 | 53.191 | Mdx | Average | 59.196 | 30.895 | 21.716 | 31.475 | 13.066 | 414.907 | 18.505 | 27.134 | 57.330 | 19.080 | 0.334 |
| | | SEM | 0.034 | 0.068 | 0.026 | 0.032 | 0.052 | 0.068 | 0.017 | 4.188 | 0.634 | | SEM | 26.691 | 16.347 | 0.042 | 1.263 | 0.692 | 1.588 | 1.448 | 0.142 | 1.766 | 1.947 | | SEM | 2.294 | 1.583 | 1.473 | 1.580 | 0.724 | 7.808 | 0.888 | 0.652 | 1.043 | 0.648 | 0.015 |
| 12 Week | | | | | | | | | | | | 12 Week | | | | | | | | | | | | 12 Week | | | | | | | | | | | | |
| WT D2 | 6 | Average | 0.965 | 1.433 | 0.872 | 1.145 | 3.707 | 2.567 | 0.495 | 101.405 | 22.183 | WT D2 | Average | 628.920 | 468.137 | 1.365 | 18.392 | 14.127 | 22.118 | 25.638 | 0.975 | 29.404 | 59.375 | WT D2 | Average | 58.173 | 31.018 | 25.403 | 33.972 | 16.060 | 464.520 | 25.005 | 26.557 | 52.078 | 17.310 | 0.334 |
| | | SEM | 0.022 | 0.088 | 0.053 | 0.040 | 0.065 | 0.201 | 0.009 | 6.834 | 0.753 | | SEM | 18.227 | 24.092 | 0.096 | 1.890 | 0.968 | 1.689 | 3.459 | 0.196 | 2.699 | 2.705 | | SEM | 6.078 | 4.301 | 4.609 | 2.837 | 2.116 | 25.979 | 1.681 | 1.425 | 3.051 | 1.039 | 0.015 |
| Sgcg -/- D2 | 13 | Average | 0.866 | 1.294 | 0.803 | 1.251 | 3.094 | 1.822 | 0.550 | 65.488 | 19.120 | KO D2 | Average | 649.947 | 466.595 | 1.404 | 19.486 | 12.855 | 18.756 | 25.280 | 0.773 | 36.688 | 39.648 | KO D2 | Average | 74.471 | 43.539 | 11.029 | 28.619 | 14.006 | 489.470 | 24.139 | 27.632 | 48.984 | 15.746 | 0.323 |
| | | SEM | 0.032 | 0.055 | 0.032 | 0.053 | 0.082 | 0.112 | 0.028 | 3.218 | 0.734 | | SEM | 19.173 | 15.901 | 0.034 | 0.963 | 0.409 | 1.054 | 1.323 | 0.054 | 2.208 | 2.552 | | SEM | 2.492 | 2.394 | 1.584 | 1.275 | 0.703 | 11.689 | 1.000 | 0.808 | 1.164 | 0.367 | 0.007 |
| Mdx | 10 | Average | 0.832 | 1.203 | 0.748 | 1.011 | 3.755 | 2.678 | 0.423 | 81.298 | 26.780 | Mdx | Average | 705.015 | 437.715 | 1.722 | 26.893 | 18.766 | 20.955 | 23.267 | 0.945 | 33.978 | 62.497 | Mdx | Average | 56.819 | 29.458 | 27.045 | 35.452 | 14.434 | 403.462 | 20.601 | 26.943 | 61.031 | 19.040 | 0.312 |
| | | SEM | 0.039 | 0.062 | 0.045 | 0.060 | 0.062 | 0.077 | 0.024 | 5.294 | 1.306 | | SEM | 24.566 | 41.350 | 0.140 | 2.099 | 1.240 | 0.954 | 1.893 | 0.070 | 1.208 | 2.301 | | SEM | 2.339 | 1.573 | 1.839 | 1.762 | 1.291 | 22.157 | 1.281 | 0.661 | 2.744 | 1.041 | 0.009 |
| 16 Week | | | | | | | | | | | | 16 Week | | | | | | | | | | | | 16 Week | | | | | | | | | | | | |
| WT D2 | 10 | Average | 1.057 | 1.610 | 1.076 | 1.585 | 3.238 | 1.836 | 0.671 | 103.329 | 22.130 | WT D2 | Average | 641.327 | 520.100 | 1.251 | 17.161 | 13.995 | 18.795 | 22.711 | 0.794 | 35.950 | 44.220 | WT D2 | Average | 74.838 | 44.755 | 12.647 | 31.573 | 15.553 | 492.914 | 23.216 | 28.053 | 48.330 | 15.635 | 0.326 |
| | | SEM | 0.057 | 0.095 | 0.041 | 0.094 | 0.118 | 0.209 | 0.040 | 5.008 | 0.713 | | SEM | 30.828 | 32.585 | 0.051 | 1.104 | 0.710 | 1.613 | 1.842 | 0.053 | 2.973 | 3.963 | | SEM | 4.862 | 4.162 | 3.471 | 1.723 | 1.115 | 23.219 | 1.683 | 1.158 | 2.085 | 0.811 | 0.016 |
| Sgcg -/- D2 | 5 | Average | 0.924 | 1.376 | 0.802 | 1.242 | 3.174 | 1.932 | 0.547 | 74.244 | 19.760 | KO D2 | Average | 622.124 | 457.744 | 1.371 | 16.702 | 13.162 | 21.514 | 25.594 | 0.848 | 29.160 | 41.164 | KO D2 | Average | 70.740 | 39.780 | 12.662 | 28.502 | 14.220 | 500.618 | 26.596 | 25.632 | 48.718 | 16.940 | 0.347 |
| | | SEM | 0.025 | 0.043 | 0.020 | 0.060 | 0.115 | 0.190 | 0.022 | 4.673 | 0.568 | | SEM | 51.893 | 37.802 | 0.085 | 1.549 | 1.767 | 1.923 | 2.386 | 0.057 | 1.399 | 3.631 | | SEM | 4.853 | 3.901 | 3.012 | 1.336 | 0.822 | 27.891 | 2.380 | 0.802 | 3.290 | 1.425 | 0.016 |
| Mdx | 8 | Average | 0.879 | 1.298 | 0.771 | 1.070 | 3.766 | 2.631 | 0.439 | 85.791 | 27.438 | Mdx | Average | 738.816 | 468.001 | 1.607 | 25.821 | 16.664 | 22.361 | 24.240 | 0.970 | 33.676 | 63.749 | Mdx | Average | 59.229 | 31.633 | 26.589 | 37.160 | 16.479 | 443.528 | 19.611 | 29.191 | 54.059 | 17.461 | 0.324 |
| | | SEM | 0.042 | 0.068 | 0.016 | 0.059 | 0.067 | 0.157 | 0.015 | 3.908 | 1.228 | | SEM | 26.539 | 27.750 | 0.089 | 2.342 | 1.059 | 1.532 | 2.499 | 0.085 | 1.497 | 2.806 | | SEM | 4.047 | 3.299 | 3.041 | 1.454 | 0.947 | 18.762 | 1.324 | 0.836 | 1.688 | 0.586 | 0.010 |
Roberts Supplemental Figure 2 Echocardiography
